# Curious Cases of Dislocation of the Patella Outwards

**Published:** 1829-07-01

**Authors:** 


### Periscope;

OR,

### CIRCUMSPECTIVE REVIEW.

"Ore trahit quodcunque potest, atque addit acervo."

### I.

### Curious Cases of Dislocation of the Patella Outwards.

Sir Astley Cooper, in his Lectures on Surgery, remarks that, of all the dislocations of the patella, that upon the outer condyle of the femur is the most common.

"Persons who have naturally an inclination of the knee inwards are most liable to this injury, and it is usually produced by a fall at the time that the knee is turned inwards and the foot outwards, so that the action of the muscles in endeavouring to prevent the fall, draw the patella over the external condyle of the thigh bone." Sir Astley further remarks, that unless the ligament has been relaxed from previous disease, *it will be torn* in this dislocation, and thus describes the manner in which the reduction may be effected.

"Place the patient in the recumbent posture, and let the leg be raised, by lifting it at the heel, so that the extensor muscles of the thigh may be relaxed as much as possible; then press down firmly the edge of the patella, furthest from the articulation, by which the opposite edge will be raised over the condyle, when the action of the muscles will quickly restore the bone to its natural situation. The following plan was adopted by Mr. George Young, in a case of the external dislocation, which he could not succeed in reducing by other means. He placed the ankle of the limb upon his shoulder, which gave him considerable power in extending the knee-joint, when grasping the patella with the fingers of his right hand, he pressed the outer edge of the bone with the ball of his left thumb, and thus forced it into its place."

We cannot entirely agree with Sir Astley in thinking, that unless the ligamentum patellæ has been previously relaxed by disease, it must necessarily be torn, because we have witnessed more than one instance of luxation of the bone where the ligament was certainly entire, though the accident occurred in healthy individuals. We were led to transfer to our pages these remarks of the worthy and able Baronet, because they are borne out by the experience of most surgeons, and will serve to contrast the more strongly with the features of two rather extraordinary cases, one of which occurred in this metropolis, the other at Berlin, and that under circumstances singularly alike.

In No. 50 of our esteemed contemporary, the Medical Gazette, will be found a communication from Mr. Mayo respecting a case which occurred to himself and Mr. Broughton, the very intelligent surgeon of the 2nd regt. of Life-Guards. A private of the 2d Life-Guards, a stout athletic young man, was struck sharply on the right knee by the knee of another soldier, as, in the exercises, two opposite lines rode through each other. They were riding at a walk, but the soldier on the right of the patient, had spurred his horse, so that it moved forward briskly. By this accident the patella was dislocated outwards, and rested with its inner edge upon the outer surface of the external condyle, the fore part of the patella facing obliquely forwards and inwards. As the patient lay with the knee extended he experienced no pain, there was no tension of the quadriceps extensor cruris; the patella admitted of a slight degree of motion forward or backward, turning upon its inner edge which seemed caught behind the prominent margin of the articular surface of the condyle.

"We tried the following methods to reduce the dislocation. 1. *The knee remaining extended,* we pressed the outer edge of the patella downwards, forcing the bone at the same time strongly inwards. 2. Force was applied in the same manner, *the joint being rather more than half bent.* 3. We used the same sort of pressure, *beginning it while the knee was bent, and continuing it as forcibly as possible at the moment that the joint was brought to the extended position.* Bending the knee to the extent described we found gave the patient great pain, and caused the patella to face, not obliquely, but directly forwards.

"These attempts proved unavailing, and we left the patient for a time. In the afternoon we met at the Anatomical Theatre, in Great Windmill-Street, and examined the nature of the dislocation in a dissected limb, when we found, that, *upon bending the knee to the utmost,* the condyle was almost wholly drawn away from the patella. And we thought it reasonable to expect, that if the joint in our patient should be found to admit of perfect flexion, the patella would in that case, as we had seen it in the dissected limb, become disengaged from the condyle, and the dislocation be spontaneously reduced by the action of the quadriceps extensor cruris.

"We returned to the Barrack Hospital, and our patient expressed his willingness to submit to the experiment which we proposed to try. He was laid upon the left side, and his right ankle was grasped by a comrade, who, when we bade him, suddenly carried the heel back to the hip, thus bending the knee to the utmost. This motion was hardly completed when the patella audibly returned into its socket."

The method employed does much credit to the ingenuity of the gentlemen engaged as well as to their perseverance, but then if we were disposed to hint an objection, we should say that half an oz. of the liquor antimonii tartarizati might perchance have saved some pain and trouble to all the parties engaged. One would think that the sudden and violent flexion of the knee-joint under such circumstances must be not unlikely to rupture the ligament of the patella or the patella itself, and we cannot but consider it a hazardous experiment. However, we would not be thought to cavil, and beg to compliment the surgical staff in all sincerity on the issue of their operations; finis consecrat opus.

The second case to which we have alluded, is detailed in Rust's Magazine,* by Dr. Wolff, physician to the Royal Corps of Cadets at Berlin.

A hussar of the guard, Daniel Steinbach, twenty years of age, and of powerful make, whilst quietly walking his horse, was thrown by a sudden plunge of the animal against his comrade on the left. The left knee was struck, and the pain produced in it was so severe that he could not keep his seat, and was obliged to be taken from his horse and carried forthwith to the barrack hospital hard by. On examining the limb after his arrival there was found a dislocation of the patella outwards nearly similar to that which occurred in Mr. Mayo's case; that is, the inner margin of bone was lodged in the groove between the condyles of the femur, whilst at the same time it was twisted half round, so that the anterior surface looked inwards towards the opposite limb, the posterior surface looked outwards, and the external border projected in front immediately beneath the skin. The common tendon of the quadriceps extensor cruris, and the ligamentum patellæ, were, of course, twisted inwards, in accordance with the position of the bone—the parts were not much swollen—the limb was firmly and almost immoveably extended—and, unless attempts at flexion were made, the patient experienced but little pain.

The limb was raised, and the knee-joint extended to the utmost by means of cushions, so as to relax as much as possible the extensors of the thigh, whilst a fillet was placed at the same time in the middle of the latter by which the soft parts were drawn down.

* Magazin für die gesammte Heilkunde; 27e. vol. 3e. cahier, 1828.

Dr. Wolff, then standing on the outside of the limb, grasped the patella betwixt his fingers and thumbs, and endeavoured to raise and turn the bone, but without success. Attempts were then made by flexing the joint, but they caused the most excessive pain, produced no good effect, and were therefore given up. The most extraordinary reasoning was now adopted by the surgeon, and gave birth to an equally extraordinary operation, that of dividing the tendon of the quadriceps extensor above the patella, and the ligament below it! The ratiocination we shall not insert: the operation adopted we shall.

A longitudinal incision was made through the skin, commencing about an inch above the superior border of the rotula, and extending down to the spine of the tibia. The tendon was laid bare, and divided by gentle touches of the knife at the point of its attachment to the bone, the capsule appearing untouched. It was then attempted, but without success, to reduce the luxation, and accordingly the ligamentum patellæ was next divided! The most extraordinary part of the business, however, is this, that although the patella was thus cut off from the tendon above, and the ligament below, it continued as immoveable as ever! All further manipulations were now given up, the wound united by suture and bandage, eighteen ounces of blood abstracted from the arm, and forty leeches applied to the knee, which was afterwards covered with ice. The patient was also directed to take a solution of Glauber's salt and nitre. Little sleep was obtained in the night in consequence of lancinating pains in the knee, but they grew less severe towards morning; and next day, Dec. 23d, the patient only felt them when pressure was made on the condyles of the femur. The pulse, however, had become more frequent, and another bleeding to 12 oz. was practised. In the evening he had a shivering which lasted two hours, and was followed by fever, with disturbance about the head, and very quick small pulse. He passed an indifferent night, and next morning had much fever, with the pulse small, frequent, and irregular, dry tongue, much cerebral disturbance, and prostration. No redness nor swelling were observed about the knee, but the patient complained of some pain in the line of the incision. The straps were removed, and the wound was found to have united up to its superior angle, which gave issue to a mixture of synovia and thin sanious matter. The dressings were reapplied, and the patient ordered a draught containing sulphuric acid. In the course of the day the patient improved, and on the 25th, was a great deal better, whilst the wound was found to have united throughout. There was no pain whatever in the joint, not even on pressure. He continued in this favourable state for the next few days, experiencing, however, much pain on motion of the limb, whilst the sinus at the upper end of the wound burst out afresh, and discharged some thick pus and synovia. From the 7th to the 22d of January, the patient complained of pain in the internal condyle of the femur, with redness, heat, and swelling of the part, hectic and progressive emaciation. Leeches were twice applied with mercurial inunctions, with relief to the pain; an opening was made with the nitrate of silver on the internal side of the joint, and the limb was placed on the apparatus of Santer, ("balancoire de Santer.") Pus flowed in considerable quantity from the wound, and one day, during some motions of the patient, a bleeding took place which was readily checked. An abscess now formed on the inside of the knee, which was opened, and furnished five or six ounces of pus. It was afterwards discovered that the bed of the abscess had its seat at the posterior part of the thigh, in the triangular space formed by the bifurcation of the linea aspera of the femur, and here the matter collected and maintained itself despite of every means adopted to prevent it. There was pain on moving the limb, but the form of the joint experienced little change. In March there supervened symptoms of chronic inflammation of the mucous membrane of the bowels, the hectic continued, the powers of the system were exhausted, anasarca and even ascites succeeded; and on the 18th of November, 1824, eleven months after the accident, the unfortunate patient died.

*Sectio Cadaveris.* The thoracic viscera were sound. The quantity of water in the cavity of the peritoneum was considerable—the mucous membrane of the intestines presented the marks of former inflammation — the kidneys were pale, and their mammillæ changed into a substance like fungus hæmatodes.

A little thin pus was found in the knee-joint, the capsule of which was thickened and adherent to the surrounding cellular membrane. The cartilage over the front and inferior surface of the condyles of the femur, as well as that which lines the pit between them was destroyed, but traces of it still remained at their posterior part, as well as on the condyles of the femur. The exposed surfaces of the bones were rough, but not eroded to any great extent. The patella which was also in great measure deprived of its cartilage, was much smaller in size than its fellow, and anchylosed with the femur between its condyles, precisely in the manner in which it has previously been described as dislocated. There was also a slighter anchylosis between the internal condyle of the femur and the corresponding part of the tibia. The fistulous openings which existed on the anterior and internal parts of the knee led to a channel which extended beneath the vastus internus to behind the bone. In the latter situation was found a large cavity extending from the condyles of the femur up to its middle, following the course of the linea aspera, which had mostly lost its periosteum, and was carious in several parts.

Dr. Wolff believes that, it was not the operation, but the mal-position in which the patella remained, that proved the cause of all the subsequent and fatal mischief. It is natural enough that the Doctor should hold this opinion, being himself a party concerned, but we must confess that we do not agree with him in toto. Dislocations, aye, and dislocations quite as severe as, if not severer than the one in question, are constantly occurring, and yet when the accident is not a compound one, how seldom do they produce any very bad consequences. Wounds of tendons are awkward matters at the best, but cutting across that of the quadriceps extensor, and the ligamentum patellæ to boot, with a wound into the knee-joint, as all who consider the anatomy of the parts must see it is next to impossible to avoid, is really a very serious sort of proceeding. The most extraordinary part of the business is, the patella continuing fixed after its tendinous attachments above and below were divided. To us it is really quite incomprehensible! It is obvious from the perusal of these two interesting cases, that a species of luxation of the rotula may take place, which is not described, at all events not commonly described, and further, that the difficulty of reduction is astonishingly great. On these accounts we have been induced to collate the cases in question, the only ones, as far as our reading extends, of the kind, and hope they may prove instructive and useful to our readers.

